# Trihexyphenidyl hydro­chloride: a powder diffraction study

**DOI:** 10.1107/S1600536810035294

**Published:** 2010-09-04

**Authors:** Elisabetta Maccaroni, Luciana Malpezzi, Norberto Masciocchi

**Affiliations:** a Dipartimento di Chimica, Materiali e Ingegneria Chimica "G. Natta", Politecnico di Milano, via Mancinelli, 7, 20131 Milano, Italy; b Dipartimento di Scienze Chimiche e Ambientali, Università degli Studi dell’Insubria, via Valleggio, 11, 22100 Como, Italy

## Abstract

In the cation of the title compound [systematic name: 1-(3-cyclo­hexyl-3-hy­droxy-3-phenyl­prop­yl)piperidinium chloride], C_20_H_32_NO^+^·Cl^−^, the cyclo­hexyl and piperidine rings are in chair conformations. In the crystal structure, cations and anions are linked into chains along the *c*-axis direction *via* O—H⋯Cl and N—H⋯Cl hydrogen bonds. Weak inter­molecular C—H⋯Cl inter­actions link further these chains into layers parallel to the *bc* plane. The salt, obtained from a racemic solution, was found to crystallize in the chiral *P*2_1_2_1_2 space group, indicating that, in the absence of any evident chirality-inducing process, the polycrystalline powders consist of an equivalent mixture of *R* and *S* enanti­omers, forming a racemic conglomerate.

## Related literature

For characterization of related structures, see Camerman & Camerman (1971*a* ▶, 1972*a* ▶); Codding (1986 ▶); Marubayashi *et al.* (1999 ▶). For structure–activity relationships, see Camerman & Camerman (1970 ▶, 1971*a* ▶,*b* ▶, 1972*a* ▶,*b* ▶, 1981 ▶). For the profile function, see: Cheary & Coelho (1992 ▶) and for the March–Dollase orientation correction, see: Dollase (1986 ▶).
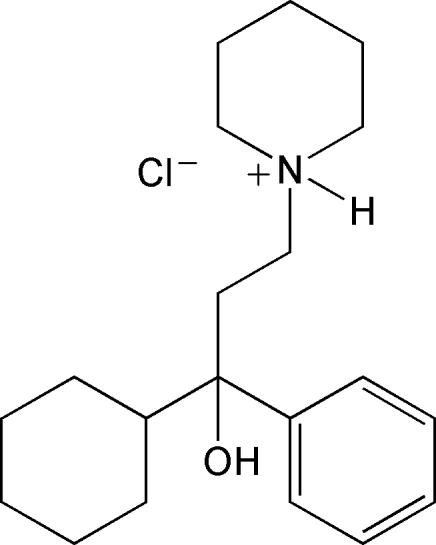


## Experimental

### 

#### Crystal data

C_20_H_32_NO^+^·Cl^−^
*M* *_r_* = 337.93
Orthorhombic, 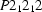

*a* = 30.0265 (8) Å
*b* = 11.2297 (4) Å
*c* = 5.8931 (2) Å
*V* = 1987.08 (12) Å^3^
*Z* = 4
Cu *K*α radiation, λ = 1.540562, 1.544390 Å
*T* = 298 K
Flat sheet, 15 × 20 mm

#### Data collection

Bruker D8 Advance diffractometer
Specimen mounting: packed powder
Data collection mode: reflection
Scan method: step
2θ_min_ = 5°, 2θ_max_ = 104.86°, 2θ_step_ = 0.02°

#### Refinement

*R*_p_ = 0.051
*R*_wp_ = 0.075
*R*_exp_ = 0.008
*R*_Bragg_ = 0.023
χ^2^ = 91.317
4994 data points
100 parameters
46 restraints
H-atom parameters constrained

### 

Data collection: *D8 Software* (Bruker, 2005 ▶); cell refinement: *TOPAS-R* (Coelho, 2005 ▶); data reduction: *TOPAS-R*; program(s) used to solve structure: *TOPAS-R*; program(s) used to refine structure: *TOPAS-R*; molecular graphics: *SHELXTL/NT* (Sheldrick, 2008 ▶); software used to prepare material for publication: *enCIFer* (Allen *et al.*, 2004 ▶).

## Supplementary Material

Crystal structure: contains datablocks global, I. DOI: 10.1107/S1600536810035294/cv2721sup1.cif

Rietveld powder data: contains datablocks I. DOI: 10.1107/S1600536810035294/cv2721Isup2.rtv

Additional supplementary materials:  crystallographic information; 3D view; checkCIF report

## Figures and Tables

**Table 1:** Hydrogen-bond geometry (Å, °)

*D*—H⋯*A*	*D*—H	H⋯*A*	*D*⋯*A*	*D*—H⋯*A*
N1—H1⋯Cl^i^	0.91	2.25	3.141 (13)	166
O1—H2⋯Cl	0.88	2.11	2.986 (13)	173
C20—H20*B*⋯Cl	0.97	2.76	3.623 (12)	149

Symmetry code: (i) 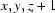.

**Fig. 1.**
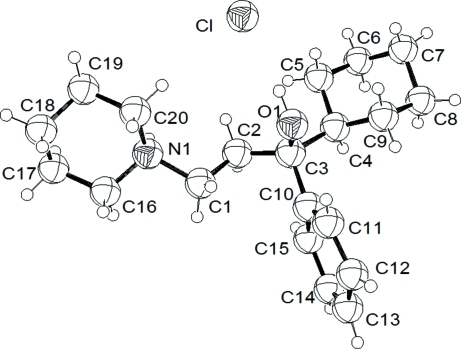
The molecular structure of 1 showing the atomic numbering and 50% probabilty displacement spheres.

**Fig. 2.**
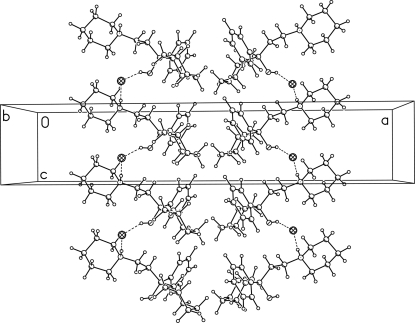
A portion of the crystal packing of 1 viewed down b axis. Intermolecular hydrogen contacts (O—H···Cl and N—H···Cl) are shown as dashed lines.

**Fig. 3.**
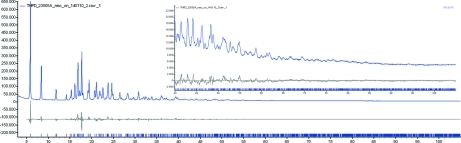
The Rietveld plot for 1 with peak markers at the bottom. The inset shows the high angle region (2θ>40°).

## supplementary crystallographic information

### Comment

The title compound, THPD-HCl (THPD = trihexyphenidyl) (1), crystallizes in the non-centrosymmetric space group *P*2_1_2_1_2, thus powders of 1 are formed by an equivalent mixture of enantiomorphic crystals in a racemic conglomerate. 1 consists of an ionic packing of N-protonated cations and chloride anions. The organic cation is formed by a phenyl and a cyclohexyl groups connected to the asymmetric carbon atom (C3) which is further linked to an hydroxyl group and to a protonated *N*-ethyl piperidine moiety (see Fig. 1). Trihexyphenidyl hydrochloride (1), is a salt used in the treatment of all forms of Parkinson's disease. Trihexyphenidyl and other pharmacological agents of the same class, such as procyclidine hydrochloride and biperiden, were completely characterized by single-crystal X-ray analyses (Camerman & Camerman, 1971*a*, 1972*a*; Codding, 1986; Marubayashi *et al.*, 1999), which showed some common stereochemical features which were correlated to their common pharmacological activity. The presence of the electron donating OH group and of the heterocyclic nitrogen has been considered as the typical stereochemical feature of these compounds.The distance between the two groups is 4.00 Å, 2.76 Å and 3.55 Å for 1, THPD, biperiden and procyclidine hydrochloride, in sequence. The shorter N—O distance observed in THPD allows the formation of intramolecular hydrogen bonds O—H···N. At variance, 1 and procyclidine hydrochloride form intermolecular hydrogen bonds through chloride anions. In the crystal, the NH group is pointing away from the direction of the OH group, allowing the formation of molecular chains running along the *c* axis, through intermolecular O—H···Cl and N—H···Cl hydrogen bonds (Table 1). Moreover, weaker C—H···Cl  interactions (Table 1) link the chains in a three-dimensional network.

### Experimental

Samples of the racemic mixture of the title compound were kindly provided by Dr. C. Pellegatta (Solmag, Divisione di Fidia Farmaceutici S.p.A., Garbagnate Milanese, Italy)

### Refinement

Approximate cell parameters for 1 were determined by the SVD indexing algorithm present in the program TOPAS-*R* (Coelho, 2005), using the first 20 peak positions, *M*(20) = 31. Structure solution was initiated by employing a semi-rigid molecular fragment (flexible about five torsion angles) taken from the known crystal structure of THPD (see Camerman & Camerman 1972*a*) and a freely floating Cl^-^ anion. Simulated annealing allowed the location and orientation of the used fragments, later refined by the Rietveld method, using the independent atom model for non-H atoms (geometrically restrained to achieve convergence to a chemically plausible structure) and idealized H-atom positions. The diffraction profile and the difference between the measured and calculated profiles are shown in Fig. 3.

### Crystal data

**Table:**

C_20_H_32_NO^+^·Cl^−^	*F*(000) = 736
*M**_r_* = 337.93	*D*_x_ = 1.130 Mg m^−^^3^
Orthorhombic, *P*2_1_2_1_2	Cu *K*α radiation, λ = 1.540562, 1.544390 Å
*a* = 30.0265 (8) Å	*T* = 298 K
*b* = 11.2297 (4) Å	Particle morphology: no specific habit
*c* = 5.8931 (2) Å	white
*V* = 1987.08 (12) Å^3^	flat sheet, 15 × 20 mm
*Z* = 4	Specimen preparation: Prepared at 298 K and 101.325 kPa

### Data collection

**Table:**

Bruker AXS D8 Advance diffractometer	Data collection mode: reflection
Radiation source: sealed X-ray tube	Scan method: step
Ni filter	2θ_min_ = 5°, 2θ_max_ = 104.86°, 2θ_step_ = 0.02°
Specimen mounting: packed powder	

### Refinement

**Table:**

Refinement on *I*_net_	Profile function: fundamental parameters (Cheary & Coelho, 1992)
Least-squares matrix: full with fixed elements per cycle	100 parameters
*R*_p_ = 0.051	46 restraints
*R*_wp_ = 0.075	H-atom parameters constrained
*R*_exp_ = 0.008	*w* = 1/σ(Y_obs_)^2^
*R*_Bragg_ = 0.023	(Δ/σ)_max_ = 0.01
χ^2^ = 91.317	Background function: Chebyshev polynomial
4994 data points	Preferred orientation correction: March–Dollase (Dollase, 1986); direction of preferred orientation 100, texture parameter *r* = 0.763).

### Special details

**Table:**

Geometry. Bond distances, angles *etc*. have been calculated using the rounded fractional coordinates. All e.s.d.'s are estimated from the variances of the (full) variance-covariance matrix. The cell e.s.d.'s are taken into account in the estimation of distances, angles and torsion angles

### Fractional atomic coordinates and isotropic or equivalent isotropic displacement parameters (Å^2^)

**Table:**

	*x*	*y*	*z*	*U*_iso_*/*U*_eq_	
O1	−0.1539 (3)	0.9849 (13)	−0.521 (2)	0.0703 (16)*	
N1	−0.2407 (2)	1.0155 (11)	−0.0092 (18)	0.0703 (16)*	
C1	−0.1953 (3)	1.0772 (12)	−0.079 (3)	0.0703 (16)*	
C2	−0.1630 (3)	0.9800 (12)	−0.118 (2)	0.0703 (16)*	
C3	−0.1306 (3)	0.9977 (10)	−0.3049 (17)	0.0703 (16)*	
C4	−0.0896 (3)	0.9088 (8)	−0.3708 (19)	0.0703 (16)*	
C5	−0.1053 (3)	0.7793 (9)	−0.349 (2)	0.0703 (16)*	
C6	−0.0643 (3)	0.6928 (9)	−0.3714 (19)	0.0703 (16)*	
C7	−0.0458 (3)	0.7068 (9)	−0.6064 (19)	0.0703 (16)*	
C8	−0.0271 (3)	0.8346 (9)	−0.617 (2)	0.0703 (16)*	
C9	−0.0682 (3)	0.9211 (9)	−0.612 (2)	0.0703 (16)*	
C10	−0.1089 (3)	1.1202 (8)	−0.2851 (17)	0.0703 (16)*	
C11	−0.1119 (4)	1.2011 (9)	−0.4647 (19)	0.0703 (16)*	
C12	−0.0882 (3)	1.3065 (10)	−0.4550 (18)	0.0703 (16)*	
C13	−0.0620 (4)	1.3275 (10)	−0.2593 (16)	0.0703 (16)*	
C14	−0.0603 (4)	1.2490 (9)	−0.0726 (18)	0.0703 (16)*	
C15	−0.0837 (4)	1.1401 (9)	−0.0896 (18)	0.0703 (16)*	
C16	−0.2651 (3)	1.0901 (10)	0.1634 (19)	0.0703 (16)*	
C17	−0.3081 (3)	1.0375 (12)	0.2536 (16)	0.0703 (16)*	
C18	−0.3374 (3)	1.0063 (13)	0.051 (2)	0.0703 (16)*	
C19	−0.3122 (3)	0.9158 (10)	−0.091 (2)	0.0703 (16)*	
C20	−0.2705 (3)	0.9634 (13)	−0.1905 (16)	0.0703 (16)*	
Cl	−0.22190 (13)	0.8068 (5)	−0.6675 (10)	0.0703 (16)*	
H1	−0.23187	0.95067	0.07164	0.0703*	
H1A	−0.19914	1.12387	−0.21648	0.0703*	
H1B	−0.18491	1.12941	0.04069	0.0703*	
H2	−0.17234	0.92801	−0.56039	0.0703*	
H2A	−0.17952	0.90757	−0.14864	0.0703*	
H2B	−0.14641	0.96752	0.02116	0.0703*	
H4	−0.06586	0.92116	−0.25892	0.0703*	
H5A	−0.12688	0.76178	−0.46648	0.0703*	
H5B	−0.11951	0.76773	−0.20272	0.0703*	
H6A	−0.04184	0.71266	−0.25946	0.0703*	
H6B	−0.07368	0.61116	−0.34674	0.0703*	
H7A	−0.06895	0.69573	−0.71908	0.0703*	
H7B	−0.02234	0.64909	−0.63362	0.0703*	
H8A	−0.01016	0.84599	−0.75576	0.0703*	
H8B	−0.00772	0.84971	−0.48882	0.0703*	
H9A	−0.05872	1.00254	−0.63816	0.0703*	
H9B	−0.08956	0.89932	−0.72825	0.0703*	
H11	−0.12967	1.18414	−0.58978	0.0703*	
H12	−0.08933	1.36158	−0.57273	0.0703*	
H13	−0.04506	1.39670	−0.25339	0.0703*	
H14	−0.04432	1.26822	0.05746	0.0703*	
H15	−0.08230	1.08356	0.02551	0.0703*	
H16A	−0.27176	1.16675	0.09554	0.0703*	
H16B	−0.24530	1.10423	0.29060	0.0703*	
H17A	−0.32313	1.09468	0.35033	0.0703*	
H17B	−0.30188	0.96662	0.34216	0.0703*	
H18A	−0.36547	0.97302	0.10171	0.0703*	
H18B	−0.34357	1.07706	−0.03843	0.0703*	
H19A	−0.30505	0.84757	0.00304	0.0703*	
H19B	−0.33137	0.88837	−0.21298	0.0703*	
H20A	−0.27764	1.02447	−0.30103	0.0703*	
H20B	−0.25477	0.89994	−0.26842	0.0703*	

### Geometric parameters (Å, °)

**Table:**

O1—C3	1.460 (15)	C2—H2A	0.9696
O1—H2	0.8767	C2—H2B	0.9696
N1—C1	1.584 (13)	C4—H4	0.9808
N1—C20	1.511 (14)	C5—H5A	0.9684
N1—C16	1.508 (15)	C5—H5B	0.9705
N1—H1	0.9096	C6—H6A	0.9694
C1—C2	1.478 (17)	C6—H6B	0.9700
C2—C3	1.483 (14)	C7—H7A	0.9693
C3—C4	1.632 (14)	C7—H7B	0.9704
C3—C10	1.527 (14)	C8—H8A	0.9714
C4—C5	1.534 (13)	C8—H8B	0.9685
C4—C9	1.566 (16)	C9—H9A	0.9701
C5—C6	1.574 (13)	C9—H9B	0.9697
C6—C7	1.500 (15)	C11—H11	0.9297
C7—C8	1.542 (14)	C12—H12	0.9301
C8—C9	1.571 (13)	C13—H13	0.9294
C10—C11	1.398 (14)	C14—H14	0.9297
C10—C15	1.396 (15)	C15—H15	0.9301
C11—C12	1.382 (15)	C16—H16A	0.9700
C12—C13	1.416 (15)	C16—H16B	0.9698
C13—C14	1.411 (15)	C17—H17A	0.9700
C14—C15	1.414 (15)	C17—H17B	0.9700
C16—C17	1.516 (14)	C18—H18A	0.9693
C17—C18	1.524 (15)	C18—H18B	0.9713
C18—C19	1.518 (17)	C19—H19A	0.9697
C19—C20	1.482 (14)	C19—H19B	0.9710
C1—H1A	0.9718	C20—H20A	0.9698
C1—H1B	0.9688	C20—H20B	0.9704
			
Cl···C20	3.623 (12)	H2B···C15	2.7799
Cl···O1	2.986 (13)	H2B···H1	2.5900
Cl···N1^i^	3.141 (13)	H2B···H9B^iii^	2.3837
Cl···C16^i^	3.577 (12)	H2B···H15	2.3248
Cl···H20B	2.7567	H4···C15	2.7069
Cl···H16A^ii^	2.9787	H4···H6A	2.4500
Cl···H5A	3.1305	H4···H8B	2.3510
Cl···H17B^i^	2.9986	H4···H15	2.5257
Cl···H1^i^	2.2501	H5A···Cl	3.1305
Cl···H2	2.1132	H5A···H9B	2.4538
O1···Cl	2.986 (13)	H5A···H17A^iv^	2.4986
O1···H9B	2.4795	H5A···H7A	2.4065
O1···H11	2.3874	H5A···O1	2.6532
O1···H2B^i^	2.7145	H5A···H2	2.3779
O1···H5A	2.6532	H5B···H2A	2.4113
O1···H1A	2.7389	H5B···C2	2.7635
N1···Cl^iii^	3.141 (13)	H6A···H4	2.4500
C1···C15	3.425 (15)	H6A···H8B	2.2902
C9···C11	3.516 (15)	H7A···H5A	2.4065
C11···C9	3.516 (15)	H7A···H9B	2.3691
C15···C1	3.425 (15)	H8A···H14^vii^	2.3521
C16···Cl^iii^	3.577 (12)	H8A···C14^vii^	3.0168
C17···C20^iii^	3.564 (14)	H8B···H4	2.3510
C20···Cl	3.623 (12)	H8B···H6A	2.2902
C20···C17^i^	3.564 (14)	H9A···C11	2.9269
C1···H19A^iv^	3.0690	H9A···C10	2.8888
C2···H5B	2.7635	H9A···H15^i^	2.2929
C2···H20B	3.0310	H9B···H5A	2.4538
C2···H15	2.8176	H9B···H7A	2.3691
C4···H15	3.0584	H9B···O1	2.4795
C5···H2	2.8970	H9B···H2B^i^	2.3837
C5···H17A^iv^	2.9860	H9B···H15^i^	2.5364
C5···H2A	2.9043	H11···O1	2.3874
C9···H15^i^	2.8409	H14···H8A^vi^	2.3521
C10···H9A	2.8888	H15···C4	3.0584
C10···H1A	2.7400	H15···C9^iii^	2.8409
C10···H1B	2.9842	H15···H2B	2.3248
C11···H9A	2.9269	H15···H4	2.5257
C13···H18A^v^	2.8767	H15···H9A^iii^	2.2929
C14···H8A^vi^	3.0168	H15···H9B^iii^	2.5364
C15···H4	2.7069	H15···C2	2.8176
C15···H2B	2.7799	H16A···Cl^viii^	2.9787
C17···H20A^iii^	2.7832	H16A···H18B	2.5072
C20···H17B^i^	2.9110	H16B···H1B	2.3531
C20···H2A	2.8138	H17A···H5A^v^	2.4986
H1···H2B	2.5900	H17A···C5^v^	2.9860
H1···H19A	2.5165	H17A···H20A^iii^	2.5901
H1···H2A	2.0953	H17B···Cl^iii^	2.9986
H1···Cl^iii^	2.2501	H17B···C20^iii^	2.9110
H1A···O1	2.7389	H17B···H19A	2.4063
H1A···C10	2.7400	H17B···H20A^iii^	2.3180
H1B···C10	2.9842	H18A···C13^iv^	2.8767
H1B···H19A^v^	2.4818	H18B···H20A	2.5811
H1B···H16B	2.3531	H18B···H16A	2.5072
H2···C5	2.8970	H19A···C1^iv^	3.0690
H2···Cl	2.1132	H19A···H1	2.5165
H2···H2A	2.4468	H19A···H17B	2.4063
H2···H5A	2.3779	H19A···H1B^iv^	2.4818
H2A···C5	2.9043	H20A···H17B^i^	2.3180
H2A···H5B	2.4113	H20A···H18B	2.5811
H2A···H20B	2.3689	H20A···C17^i^	2.7832
H2A···C20	2.8138	H20A···H17A^i^	2.5901
H2A···H1	2.0953	H20B···Cl	2.7567
H2A···H2	2.4468	H20B···C2	3.0310
H2B···O1^iii^	2.7145	H20B···H2A	2.3689
			
C3—O1—H2	127.25	C5—C6—H6B	110.09
C1—N1—C16	110.5 (10)	C7—C6—H6A	110.27
C16—N1—C20	113.8 (7)	C7—C6—H6B	110.17
C1—N1—C20	119.7 (10)	H6A—C6—H6B	108.52
C20—N1—H1	103.47	C6—C7—H7A	110.70
C1—N1—H1	103.61	C6—C7—H7B	110.57
C16—N1—H1	103.48	C8—C7—H7A	110.65
N1—C1—C2	106.4 (10)	C8—C7—H7B	110.52
C1—C2—C3	116.5 (11)	H7A—C7—H7B	108.76
O1—C3—C4	95.4 (8)	C7—C8—H8A	110.32
O1—C3—C10	111.1 (9)	C7—C8—H8B	110.50
C2—C3—C4	126.1 (9)	C9—C8—H8A	110.22
C2—C3—C10	110.1 (9)	C9—C8—H8B	110.43
O1—C3—C2	108.7 (8)	H8A—C8—H8B	108.60
C4—C3—C10	104.3 (7)	C4—C9—H9A	110.35
C3—C4—C5	109.2 (7)	C4—C9—H9B	110.34
C3—C4—C9	118.1 (8)	C8—C9—H9A	110.45
C5—C4—C9	106.6 (8)	C8—C9—H9B	110.51
C4—C5—C6	109.7 (7)	H9A—C9—H9B	108.63
C5—C6—C7	107.6 (8)	C10—C11—H11	120.28
C6—C7—C8	105.6 (9)	C12—C11—H11	120.24
C7—C8—C9	106.8 (7)	C11—C12—H12	121.32
C4—C9—C8	106.5 (8)	C13—C12—H12	121.14
C11—C10—C15	123.8 (9)	C12—C13—H13	118.29
C3—C10—C11	120.0 (9)	C14—C13—H13	118.26
C3—C10—C15	116.0 (8)	C13—C14—H14	121.10
C10—C11—C12	119.5 (10)	C15—C14—H14	121.04
C11—C12—C13	117.5 (10)	C10—C15—H15	121.13
C12—C13—C14	123.5 (10)	C14—C15—H15	121.09
C13—C14—C15	117.9 (10)	N1—C16—H16A	108.37
C10—C15—C14	117.8 (9)	N1—C16—H16B	108.33
N1—C16—C17	115.7 (9)	C17—C16—H16A	108.34
C16—C17—C18	107.9 (8)	C17—C16—H16B	108.35
C17—C18—C19	107.3 (8)	H16A—C16—H16B	107.45
C18—C19—C20	113.4 (10)	C16—C17—H17A	110.15
N1—C20—C19	111.1 (8)	C16—C17—H17B	110.15
N1—C1—H1A	110.51	C18—C17—H17A	110.12
N1—C1—H1B	110.66	C18—C17—H17B	110.12
C2—C1—H1A	110.28	H17A—C17—H17B	108.46
C2—C1—H1B	110.41	C17—C18—H18A	110.43
H1A—C1—H1B	108.59	C17—C18—H18B	110.31
C1—C2—H2A	108.23	C19—C18—H18A	110.20
C1—C2—H2B	108.19	C19—C18—H18B	110.09
C3—C2—H2A	108.04	H18A—C18—H18B	108.47
C3—C2—H2B	108.10	C18—C19—H19A	108.92
H2A—C2—H2B	107.39	C18—C19—H19B	108.96
C3—C4—H4	107.56	C20—C19—H19A	108.91
C5—C4—H4	107.53	C20—C19—H19B	108.80
C9—C4—H4	107.42	H19A—C19—H19B	107.67
C4—C5—H5A	109.77	N1—C20—H20A	109.39
C4—C5—H5B	109.65	N1—C20—H20B	109.31
C6—C5—H5A	109.75	C19—C20—H20A	109.50
C6—C5—H5B	109.62	C19—C20—H20B	109.48
H5A—C5—H5B	108.29	H20A—C20—H20B	108.00
C5—C6—H6A	110.19		
			
C16—N1—C1—C2	144.9 (11)	C2—C3—C10—C11	−122.4 (10)
C20—N1—C1—C2	−79.8 (14)	C3—C4—C5—C6	−170.5 (8)
C1—N1—C16—C17	−176.9 (9)	C3—C4—C9—C8	174.3 (8)
C20—N1—C16—C17	45.0 (14)	C5—C4—C9—C8	−62.5 (9)
C1—N1—C20—C19	−177.0 (10)	C9—C4—C5—C6	60.9 (10)
C16—N1—C20—C19	−43.2 (14)	C4—C5—C6—C7	−64.1 (11)
N1—C1—C2—C3	143.5 (10)	C5—C6—C7—C8	66.4 (9)
C1—C2—C3—O1	−72.9 (14)	C6—C7—C8—C9	−69.6 (10)
C1—C2—C3—C4	175.4 (10)	C7—C8—C9—C4	67.6 (10)
C1—C2—C3—C10	49.0 (12)	C3—C10—C11—C12	−173.1 (9)
O1—C3—C4—C5	−77.8 (10)	C15—C10—C11—C12	1.4 (16)
C2—C3—C10—C15	62.7 (11)	C3—C10—C15—C14	175.3 (9)
C4—C3—C10—C11	99.8 (10)	C11—C10—C15—C14	0.6 (16)
C4—C3—C10—C15	−75.2 (11)	C10—C11—C12—C13	−0.5 (15)
C10—C3—C4—C5	168.8 (8)	C11—C12—C13—C14	−2.4 (16)
C10—C3—C4—C9	−69.3 (10)	C12—C13—C14—C15	4.4 (17)
O1—C3—C10—C11	−1.9 (13)	C13—C14—C15—C10	−3.4 (16)
O1—C3—C10—C15	−176.8 (9)	N1—C16—C17—C18	−53.6 (13)
O1—C3—C4—C9	44.1 (11)	C16—C17—C18—C19	60.4 (13)
C2—C3—C4—C5	40.0 (13)	C17—C18—C19—C20	−64.4 (12)
C2—C3—C4—C9	161.9 (9)	C18—C19—C20—N1	54.5 (13)

Symmetry codes: (i) *x*, *y*, *z*−1; (ii) ; (iii) *x*, *y*, *z*+1; (iv) ; (v) ; (vi) −*x*, −*y*+2, *z*+1; (vii) −*x*, −*y*+2, *z*−1; (viii) .

### Hydrogen-bond geometry (Å, °)

**Table:**

*D*—H···*A*	*D*—H	H···*A*	*D*···*A*	*D*—H···*A*
N1—H1···Cl^iii^	0.91	2.25	3.141 (13)	166
O1—H2···Cl	0.88	2.11	2.986 (13)	173
C11—H11···O1	0.93	2.39	2.756 (17)	103
C20—H20B···Cl	0.97	2.76	3.623 (12)	149

Symmetry codes: (iii) *x*, *y*, *z*+1.
